# Profiles of Physical Activity and Physical Performance in Matched Religious Vegetarian and Nonvegetarian Women: A Preliminary Observational Study in Taiwan

**DOI:** 10.3390/nu14102170

**Published:** 2022-05-23

**Authors:** Yu-Zu Wu, Yun-Ting Chan, Jyh-Gang Hsieh, Jia-Ching Chen

**Affiliations:** 1 Department of Physical Therapy, College of Medicine, Tzu Chi University, Hualien 97004, Taiwan; omgsandy0130@gmail.com (Y.-T.C.); c123010@mail.tcu.edu.tw (J.-C.C.); 2 Department of Rehabilitation Medicine, Hualien Tzu Chi Hospital, Buddhist Tzu Chi Medical Foundation, Hualien 97002, Taiwan; 3 School of Medicine, Tzu Chi University, Hualien 97004, Taiwan; jyhgang@gmail.com; 4 Department of Family Medicine, Hualien Tzu Chi Hospital, Buddhist Tzu Chi Medical Foundation, Hualien 97002, Taiwan

**Keywords:** Buddhist, meatless, physical activity, physical performance, religious, vegetarians, women

## Abstract

Religious vegetarianism has become more popular with women and increases with age. However, concerns have been raised that vegetarians are less productive than nonvegetarians. Thus, we aimed to compare the characteristics of physical activity and physical performance in properly matched religious vegetarian and nonvegetarian women aged ≥ 45 years. Participants (*n* = 160) were recruited via convenience sampling in the community of Hualien, Taiwan, and matched by demographic and cognitive characteristics. Physical activity was assessed using the International Physical Activity Questionnaire-Short Form (IPAQ-SF). Physical performance was assessed with handgrip strength, five-times-sit-to-stand, gait speed, timed up-and-go, and functional reach tests (FRT). Overall, 90% of religious vegetarians practiced lacto-ovo-vegetarianism. The proportions of those with low physical activity levels and poor physical performance did not significantly differ between religious vegetarians and nonvegetarians. Additionally, there were no significant between-group differences in IPAQ-SF scores and physical performance, except for FRT performance (mean 24.5 cm vs. 19.7 cm, *p* < 0.001). Exhaustion after work, busyness, and a lack of interest were three main reasons for low physical activity levels, and none of these had significant between-group differences (*p* = 0.936). Our results show a similar profile of physical activity and physical performance in religious vegetarian and nonvegetarian women.

## 1. Introduction

Vegetarianism has become increasingly popular worldwide because of religious beliefs, ethical motivations, animal welfare, health, and environmental issues [1,2,3,4]. In Asia, the practice of vegetarianism is usually linked to religious traditions of not killing living beings, e.g., Buddhism [3]. Although evidence suggests that vegetarians are often healthier, with a lower risk of obesity, high blood pressure, diabetes, and some types of cancer than nonvegetarians [5,6,7,8], there are still some concerns that vegetarians may have lower levels of physical activity and poorer physical performance, which would reduce their capacity to carry out activities of daily living and may therefore be less productive [1]. Low physical activity has been listed as one risk factor of the greatest importance for 35 chronic conditions, including sarcopenia [9], while poor physical performance has been linked to serious problems in older adults, such as cognitive dysfunction, high risk of falls, and poor quality of life [10], and has also been used as one of the diagnostic criteria for possible sarcopenia (an early manifestation of sarcopenia) [11]. Therefore, it is urgent to understand whether individuals adhering to vegetarianism would reduce physical activity levels and/or physical performance.

Growing studies have explored sports performance across a range of sports in vegetarian and nonvegetarian athletes [12,13,14,15,16]; however, two recent review studies investigating physical performance reported some controversial results from vegetarian and nonvegetarian athletes [15,17]. On the other hand, evidence specifically targeting vegetarians from community-dwelling general populations for insights into physical activity levels and physical performance is relatively scarce and inconclusive [18]. An Indian Migration Study in urban factory settings reported that Indian vegetarian adults engaged in less physical activity than nonvegetarian adults [19], but a cross-sectional study in Brazilian men showed that vegetarians were more physically active than nonvegetarians [20]. Additionally, a recent review study indicated that previous studies failed to demonstrate consistent differences in physical performance between vegetarians and nonvegetarians [18]. Two small sample studies in the Netherlands and the US showed that vegetarians did not show lower handgrip strength [21] and poorer aerobic capacity [22], respectively, in institutionalized older adults and in community-dwelling older women. More recently, two Asian cross-sectional studies have examined muscle strength in community-dwelling vegetarian and nonvegetarian older adults: one reported that there was no significant difference in biceps strength between vegetarian and nonvegetarian Chinese women [23], and the other showed that vegetarians had lower handgrip strength than nonvegetarians in Sri Lankan women [24].

Higher consumption of a healthier diet, such as vegetables and fruit, has been reported to be positively associated with better physical performance, e.g., faster gait speed and shorter duration of the timed up-and-go (TUG) and chair rise tests; however, most of these studies focused on Mediterranean diets [25,26,27,28]. For example, a recent population-based prospective cohort study in older Swedish men indicated that Mediterranean diets tended to protect against the development of low muscle mass, low muscle strength, and/or poor physical performance [27]. An analysis from two cross-sectional studies indicated that adherence to a Mediterranean diet was positively associated with gait speed in a type 2 diabetes sample of middle-aged and older adults [28]. Mediterranean diets comprise high proportions of vegetables, fruit, nuts, olive oil, and legumes; moderate amounts of fish; and low amounts of meat and their products. Similar to Mediterranean diets, vegetarian diets are also comprised of high amounts of vegetables, fruit, mushrooms, and legumes (including soybeans and soy products), but devoid of flesh foods, such as meat, poultry, wild game meat, fish, seafood, and their products [29]. To our knowledge, few studies have explored whether vegetarians specifically targeted in a sample of community-dwelling general populations are more likely to exhibit declines in physical performance than nonvegetarians.

Although higher consumption of vegetables and fruit has been linked to better muscle strength and physical performance, the relationships have gender specificity [30]. In Taiwan, approximately 25% of older adults practice vegetarianism [2], a majority are women with religious beliefs, and the proportion increases with age [2,3,31]. Taiwanese vegetarians rely highly on rice, soybeans, and their products as seafood, meat, and poultry alternatives [32], as opposed to the milk and cheese typical of Western countries. It also differs from the Mediterranean diet of properly consuming fish. Considering dietary structures vary across territories of the world, as well as there is growing interest and practice in becoming vegetarians, particularly in Taiwanese women, it is urgent to understand whether strict adherence to religious vegetarianism among Taiwanese women would decrease physical activity and physical performance. In this work, we hypothesize that middle-aged and older Taiwanese women strictly adhering to religious vegetarianism (meatless diets) have low levels of physical activity and poor physical performance. Thus, we conducted a matched sampling study aimed at exploring the differences in physical activity and physical performance in comparable groups of religious vegetarians and nonvegetarians in a sample of community-dwelling middle-aged and older Taiwanese women.

## 2. Materials and Methods

### 2.1. Study Design and Participants

This preliminary observational study by convenience sampling was conducted from February 2021 to January 2022 in Hualien City, Taiwan. Inclusion criteria included being community-dwelling women aged 45 years and over and being able to provide written informed consent. Exclusion criteria included having a cognitive impairment, as defined by a score of < 21–24 on the Mini Mental State Examination (MMSE) according to educational attainment [33], presenting with neuromuscular, musculoskeletal, or other medical conditions limiting mobility, or having an acute illness, fracture, or surgery within the past 6 months. Since no register of vegetarians exists, religious vegetarians were mostly recruited from Tzu Chi volunteers through the help of the Buddhist Tzu Chi Foundation. The Buddhist Tzu Chi Foundation is an international non-profit, non-governmental, humanitarian organization founded in 1966 in Taiwan that promotes a vegetarian (meatless) lifestyle, which is strongly influenced by Buddhist philosophies and precepts, i.e., not to kill living beings. Nonvegetarians were recruited from community development associations or local community centers and matched according to age. In the present study, a four-question questionnaire was used to identify dietary behaviors: “Are you consuming a vegetarian diet, i.e., a plant-based and meatless diet, such as rice, wheat, vegetables, fruit, nuts, mushrooms, and legumes (including soybeans and soy products)? [2]” “Are your daily diets devoid of any flesh foods, such as meat, poultry, wild game meat, fish, seafood, mollusks, crustaceans, and their products? [2]” “Are you eating eggs and dairy products?” “Have you been following the dietary patterns every day for the past 6 months?” Religious vegetarians were defined as those who had strictly eaten no flesh foods (except eggs and dairy products) for religious reasons for at least 6 months before enrolling in the study. In the present study, lacto-vegetarians (consuming meatless foods and dairy), ovo-vegetarians (consuming meatless foods and eggs), lacto-ovo-vegetarians (consuming meatless foods, eggs, and dairy), and vegans were collectively referred to as religious vegetarians. Matched nonvegetarians were operationally defined as those consuming any type of flesh foods within the past 6 months, including usually eating flesh foods (omnivores), eating small amounts of flesh foods (flexitarians or semi-vegetarians), or no meat but fish (pesco-vegetarians) [34], as well as those only eating vegetarian diets at breakfast or on the first and fifteenth days of the lunar month.

Participants were also asked to self-report information regarding their history of falls and comorbidities. A history of falls was defined as experiencing any fall event over the past 6 months. Comorbidities were assessed based on self-reported physician diagnoses and included hypertension, cardiac disease, diabetes mellitus, hyperlipidemia, and osteoarthritis [3]. The study was conducted in accordance with the Declaration of Helsinki, and the protocol was approved by the Research Ethics Committee of the Hualien Tzu Chi Hospital (IRB110-009-B). All participants gave written informed consent for inclusion in the study.

### 2.2. Sample Size

The sample size of the study was estimated a priori using previously published data on the standard deviation of gait speed from dietary habits in community-dwelling older Taiwanese women [35]. The calculated total number of 160 (80 per group) would be sufficient to detect a difference between conditions at a power of 95% and a significance level of α = 0.05, considering 10% of possible data loss.

### 2.3. Physical Activity Measures

Physical activity levels were assessed using a Taiwanese version of the International Physical Activity Questionnaire-Short Form (IPAQ-SF) by a licensed physical therapist. Using the Taiwanese version of the IPAQ-SF was permitted by the Health Promotion Administration at the Ministry of Health and Welfare in Taiwan. It has been shown to have adequate test-retest reliability, content validity, and consistency values [36]. Participants were asked to report four specific activity types in the last 7 days, including vigorous-intensity activities (e.g., running and aerobics), moderate-intensity activities (e.g., leisure cycling), walking, and sitting that are undertaken during work, transport, housework, or leisure activities. The total score is the summation of the duration and frequency of walking, moderate-, and vigorous-intensity activities, which was reported as the “metabolic equivalent of task-min per week (MET-minute/week)”. The IPAQ-SF scores of <600, 600–3000, and > 3000 MET-min/week were respectively categorized as low, moderate, and high physical activity levels according to the guidelines of the IPAQ [37]. Participants categorized as having low levels of physical activity were asked to give their reasons via spontaneous answers.

### 2.4. Physical Performance Measures

Handgrip strength, the five-times-sit-to-stand (5xSTS) test, gait speed, the TUG test, and the functional reach test (FRT) involving domains across strength, mobility, and agility/dynamic balance were adopted to assess physical performance, all of which were conducted by a licensed physical therapist. Handgrip strength was measured twice in a seated position (with an arm 90 degrees to the side). Participants were instructed to squeeze the handle of the Jamar hydraulic handheld dynamometer (Model 5030; Sammons Preston, IL, USA) as hard as possible using the dominant hand with a 30 s break between trials [11]. The maximum value was recorded for further analysis. The 5xSTS performance was assessed twice by asking participants to stand and sit five times as quickly as possible twice, with arms folded across the chest. The minimum time needed to complete the test was recorded. Gait speed was measured twice using the usual and comfortable pace along a 10 m walkway. The time required to complete the walking task was computed and averaged. Those with handgrip strength < 18 kg, 5xSTS ≥ 12 s, or gait speed < 1.0 m/s are recognized as having low physical performance [11]. The TUG was measured by standing up, walking at a comfortable pace to a cone on the floor 3 m away, turning around, and then returning to the seated position in the chair, after which the average time over two trials was calculated. Those with a TUG of > 11 s were recognized as having a risk of future fall(s) in community-dwelling older adults [38]. The FRT was measured in a standing position (with shoulders flexed 90 degrees and elbows and hands outstretched) by reaching horizontally forward as far as possible without losing balance. The maximum reach distance was recorded and then averaged over three trials for further analysis. Those with an FRT of <24.5 cm were recognized as having a high risk of recurrent falls in community-dwelling older adults [39].

### 2.5. Statistical Analysis

Both descriptive and inferential statistics were employed for data analysis. For the categorical variables, frequencies and percentages were used as descriptive measures. The chi-square test or the Fisher exact test was utilized to compare differences in frequency distributions between vegetarian and nonvegetarian groups. For the continuous variables, data were first checked for normality within groups using the Kolmogorov–Smirnov test. If data were normally distributed, the independent *t*-test was utilized to determine between-group differences, and the mean and 95% confidence intervals (CI) were used to characterize the results; however, if data were not normally distributed, the Mann–Whitney test was applied, and the median and interquartile range (IQR) were used to present the results. Analysis of covariance (ANCOVA) was used to adjust for baseline measures as a covariate and to provide an unbiased estimate of the mean difference between vegetarian and nonvegetarian groups. SPSS version 20.0 (IBM, Armonk, NY, USA) was used for statistical analysis. A probability value of *p* < 0.05 was considered statistically significant.

## 3. Results

A total of 160 community-dwelling women aged 45–89 years (median 64.5 years), with a median BMI of 23.2, were recruited in Hualien City, Taiwan. Overall, 90% of religious vegetarians (*n* = 72) practiced lacto-ovo-vegetarianism. The participant data are presented in Table 1, showing that except for osteoarthritis (*p* = 0.035), age, BMI, MMSE, educational levels, living arrangements, comorbidities, time spent sitting, and a history of falls did not show significant differences between vegetarians and nonvegetarians (*p* > 0.05, for all).

Table 2 displays the differences in physical activity and physical performance between vegetarians and nonvegetarians, showing that only the FRT value was significantly higher in vegetarians than in nonvegetarians (mean 24.5 cm vs. 19.7 cm, *p* < 0.001). There were no between-group differences regarding IPAQ-SF scores (*p* = 0.105), gait speed (*p* = 0.991), TUG (*p* = 0.676), handgrip strength (*p* = 0.932), and 5xSTS (*p* = 0.895). After adjusting for osteoarthritis as a covariate, there was still a significantly higher FRT value in vegetarians than in nonvegetarians (*p* < 0.001).

Table 3 shows the numbers (percentages) of vegetarians and nonvegetarians with low physical activity levels and poor physical performance, indicating that there were no significant between-group differences regarding low physical activity levels (*p* = 0.490) and poor physical performance (*p* > 0.05, for all).

Table 4 presents the main reasons for low physical activity levels, showing overall, 30% (48 out of 160) of participants had low physical activity levels (IPAQ-SF score of <600 MET-min/week) with four main reasons, i.e., exhaustion after work (35.4%), busyness or a lack of time (27.1%), a lack of interest (20.8%), and knee pain (16.7%). There were no significant differences in the four main causes for low physical activity levels between vegetarians and nonvegetarians (*p* = 0.936).

## 4. Discussion

In the present preliminary study, we demonstrated that the proportions of religious vegetarians with low physical activity levels and poor physical performance were not significantly higher than those of nonvegetarians in a sample of community-dwelling middle-aged and older Taiwanese women. There were no significant differences in both physical activity and physical performance between religious vegetarian and nonvegetarian women, except for FRT performance. Furthermore, we identified that exhaustion after work, a lack of time (busyness), and a lack of interest were the first three main factors in response to low physical activity levels, and these factors did not show significant differences between religious vegetarian and nonvegetarian women.

Similar to previous findings from two cohort studies in France [40] and the US [41], our study did not find significant differences in the amount of physical activity or in the number of those with low physical activity levels between vegetarian and nonvegetarian women. However, our findings are inconsistent with the results of an Indian Migration study [19], which reported less physical activity by vegetarians than by nonvegetarians. This can partially be ascribed to the discrepancy in the types of vegetarians that only lacto-vegetarians were recruited in the Indian Migration study [19], whereas 90% of vegetarians enrolled in the present study practiced lacto-ovo-vegetarianism. Indeed, cross-sectional data from the Third India Family Health Survey has indicated that lacto-vegetarians have a more sedentary behavior (less physical activity levels) than lacto-ovo-vegetarians [42]. Furthermore, health, ethical, environmental, and spiritual reasons are considered the most important factors in choosing a vegetarian diet [4,43]. In India, dietary patterns are bound by religious, cultural, and family values, which have been commonly maintained for generations [19]. However, in the present study, only religious-motivated vegetarians were recruited to the study. It has been suggested that psychological and physical behaviors may differ between subtypes of vegetarians [43], e.g., healthy-motivated vs. ethical-motivated vegetarians may exhibit different levels of physical activity, and thus may create difficulties when comparing the groups of participants with different subtypes of vegetarians. On the other hand, our results are also incompatible with the findings of a Brazilian study, reporting vegetarians being more physically active than nonvegetarians [20]. One possible explanation is that physical activity is inversely associated with BMI [44]. Vegetarians enrolled in the Brazilian study had a significantly lower BMI than nonvegetarians (mean 23.1 vs. 27.2 kg/m^2^) [20], while vegetarians recruited in the present study had a similar BMI to nonvegetarians (median 23.1 vs. 23.4 kg/m^2^).

In addition to vegetarian types and BMI, sociodemographic variables and health conditions have been suggested to be associated with physical activity levels [45,46]. However, in the present study, we did not find significant differences in sociodemographic variables and comorbidities (except for osteoarthritis) between vegetarian and nonvegetarian women. Although vegetarians had a higher proportion of osteoarthritis than nonvegetarians in our sample, they did not show significant differences in physical activity levels compared to nonvegetarians. Furthermore, we found that knee pain-induced low physical activity levels were described by all of the nonvegetarians with osteoarthritis (n = 4), while those were reported by only one-third (4 out of 12) of the vegetarians. A plausible explanation is that plant-based diets naturally contain lower levels of arachidonic acid than meat diets [47], and arachidonic acid has been considered to be a precursor of inflammatory factors involved in osteoarthritis [48]. Therefore, vegetarians consuming a diet low in arachidonic acid levels would be able to reduce joint inflammation and alleviate the expression of painful osteoarthritis during exercise, and thus increase physical activity levels. This is consistent with the findings of a prospective randomized open-label trial showing that whole-food, plant-based diets can significantly improve pain and functional status in patients with osteoarthritis [47]. On the other hand, our findings did not support the idea that vegetarians would have a healthier lifestyle than nonvegetarians, e.g., more physical activity or exercise [49]. This could partially be ascribed to the vegetarians in our sample who were recruited for religious rather than health reasons. Interestingly, consistent with previous studies in community and institutional locales [50,51], approximately 30% of individuals are physically inactive. Moreover, the first three main factors for low physical activity, i.e., exhaustion after work, a lack of time (busyness), and a lack of interest, did not show significant differences between vegetarians and nonvegetarians (*p* = 0.936). Further research is required to establish the relationship between vegetarians and physical activity levels.

Although growing evidence suggests vegetarian athletes are less likely to experience poor physical performance or exercise capacity [12,13,14,15], some studies reported controversial results [16,17]. Therefore, a representative survey of general German adults has indicated that approximately 21% of vegetarians and 74% of omnivores are concerned about being less productive (poor physical performance) following a vegetarian diet [1]. Similar to previous results that diets had neither advantages nor disadvantages with regard to exercise capacity for runners [12,13,14,15], we also found that vegetarians had comparable results in handgrip strength, gait speed, and the 5xSTS and TUG tests compared to nonvegetarians, and that the numbers of those who had poor physical performance did not significantly differ between vegetarians and nonvegetarians. Our results are congruent with previous findings from two cohort studies in Asia: one reported that handgrip strength was not associated with any type of dietary pattern in older Japanese adults at 3-year follow-up [52]; and the other conducted in Hong Kong indicated that dietary patterns were not associated with muscle mass, muscle strength, and/or physical performance in older Chinese adults at 4-year follow-up [53]. One possible explanation is that Taiwanese diets (in spite of being vegetarian or nonvegetarian) are similar to traditional diets of Asian countries, consuming more rice, soybeans, wheat, and vegetables, as opposed to the flesh foods and dairy products (cheese) typical of Western countries [31]. Thus, religious vegetarians and nonvegetarians in Taiwan essentially stayed on similar dietary habits, except that religious vegetarians were completely devoid of consuming flesh foods for religious precepts (vegetarianism) [2,54]. Additionally, our findings seem to echo a 3-year cohort study in older Japanese adults, reporting that gait speed was not associated with any type of dietary patterns (e.g., Mediterranean- and Japanese-style diets) [52]. A recent systematic review has shown that evidence for an association between plant-based dietary patterns and physical performance is strong but inconclusive [55]. Indeed, physical performance has been strongly linked to age and physical activity levels [56,57,58]. However, in the present study, we did not find significant differences in both age and physical activity levels between vegetarian and nonvegetarian women.

Interestingly, only the mean FRT values, rather than the numbers of those with FRT deficits (<25.4 cm), showed significant between-group differences, i.e., vegetarian women had higher FRT values than nonvegetarian women. Our findings seem to echo the results of a cross-sectional analysis from a cohort study of middle-aged Australian women, which reported that those who consumed more plant-based diets had a tendency toward better FRT performance [59]. One possible explanation is that plant-based diet consumption was positively associated with lower-limb muscle strength [59], while lower-limb muscle strength was positively correlated with RFT performance [60]. However, in the present study, we did not find significant between-group differences in 5xSTS performance, a proxy of lower-limb muscle strength. Additionally, age and height have been suggested to influence FRT performance [61], but we did not find significant between-group differences in both age and height (Table 1). To date, only one cross-sectional analysis compared FRT performance between vegetarians and nonvegetarians [59]. More research is required to test our findings.

In the present study, we used a four-question questionnaire about whether or not to eat meat over the past 6 months to classify participants as nonvegetarians or vegetarians. Studies suggest that some people who self-defined as vegetarians also eat flesh foods as part of their diets [62,63], which can be ascribed to the discrepancy in motivations for becoming a vegetarian and in operational definitions for conducting vegetarian studies [64]. People who choose to become a vegetarian for health reasons typically have more flexibility in using animal foods and products, e.g., flexitarians [64]. Additionally, some studies operationally defined vegetarians as those who eat flesh foods less than once per week (semi-vegetarian) [28], less than one per month [8], or less than 10 g/d [62], as well as those who occasionally eat fish, but not meat or meat products (pesco-vegetarians) [65]. In the present study, participants were vegetarians due to religious reasons, e.g., the Buddhist precept of abstaining from killing any living beings, and hence they strictly abide by their religious doctrine and adhere to a meatless diet with complete avoidance of flesh foods and their products. Moreover, our study clearly provides an operational definition of nonvegetarians as omnivores, flexitarians, semi-vegetarians, and pesco-vegetarians, as well as those only eating vegetarian diets at breakfast or on the first and fifteenth days of the lunar month. Therefore, the possible bias arising from participants’ self-declaration in our sample can be reasonably eliminated.

The strength of this study is that, to our knowledge, it is the first preliminary study to compare physical activity and physical performance between religious vegetarian and nonvegetarian women aged 45 years and over. Second, the IPAQ-SF was used to assess the levels of physical activity in the present study. The advantages of using IPAQ-SF include good reliability and validity [36,66], the ability to determine discrete categories of activity level (e.g., low, moderate, or high), and provide details about the quality of physical activity (e.g., activity type, intensity, frequency, and duration) [67]. Moreover, IPAQ-SF is the most common method of physical activity assessment [66], and it facilitates comparisons with other studies. Finally, we adopted a simple dichotomy of dietary habits, i.e., vegetarians and nonvegetarians, rather than a specific food or nutrient intake because there may be complex interactions between foods [23]. The dichotomy of dietary habits can highlight the impact of overall diets on physical activity levels and physical performance, which is of particular concern for the general population adhering to or attempting to practice religious vegetarianism [1]. Furthermore, dietary habits can help prevent or reduce the probability of recall bias toward certain foods and nutrients [23,53].

However, our study has several limitations. First, convenience sampling was used in the present study. Therefore, the potential bias of selection cannot be completely ruled out because most religious vegetarians were recruited from Tzu Chi volunteers through the Buddhist Tzu Chi Foundation. Second, only religious vegetarians were recruited in the present study. This might make it difficult to compare with other studies in which vegetarians with health reasons were also included. Vegetarians have various motivations for choosing their diet; hence, future research should clarify whether certain subtypes may be more obviously associated with physical activity and physical performance [43]. Third, an in-depth dietary assessment was not used in the present study. Although advantages of the dichotomy of dietary habits have been proposed [23,53], the absence of a dietary assessment makes it impossible to further rule out the impact of possible between-group differences in caloric and nutrient intakes in our sample on physical activity and physical performance. Fourthly, mental health has been suggested to influence physical activity [68]. Information regarding the participants’ mental health conditions was not included in our sample, and thus the effects of mental health on physical activity cannot be completely eliminated, which required further research to verify. Finally, only community-dwelling women aged 45 years and over were recruited in the present study. Thus, we caution against generalizing our findings to adult men or to populations dwelling in institutional locales. Although these limitations mean that the present results must be interpreted with caution, the results justify further large-scale research of physical activity and physical performance through dietary assessment in community-dwelling middle-aged and older adults from other provinces in Taiwan.

## 5. Conclusions

Our results suggest that Taiwanese religious vegetarian (meatless) women did not show significant differences in physical activity and physical performance compared with matched nonvegetarian women. Additionally, the proportions of religious vegetarian women with low physical activity levels and poor physical performance were similar to those of matched nonvegetarian women. The implication of these findings is to overturn the common idea of vegetarians being less productive and replace it with religious vegetarians being no less productive and also having better FRT performance than nonvegetarians, especially among the lacto-ovo-vegetarian women. More longitudinal studies are needed to further clarify the effects of vegetarian diets on physical activity levels and physical performance.

## Figures and Tables

**Table 1:** Participant characteristics (*n* = 160).

Variables	Nonvegetarians (*n* = 80)	Vegetarians (*n* = 80)	*p-*Value
Age (years)	64.5 (54.3–74.8)	64.5 (53.5–74.5)	0.928 ^a^
Height (m)	1.54 (1.51–1.60)	1.55 (1.51–1.59)	0.852 ^a^
Weight (kg)	55.8 (51.0–63.4)	55.0 (51.0–61.0)	0.384 ^a^
BMI (kg/m^2^)	23.4 (21.4–26.0)	23.1 (20.8–25.2)	0.276 ^a^
MMSE (score)	29.0 (25.0–30.0)	28.0 (26.0–29.0)	0.458 ^a^
Educational levels, *n* (%)			0.402 ^b^
Elementary school or less	30 (37.5%)	22 (27.5%)	
Secondary school	30 (37.5%)	35 (43.8%)	
College or higher	20 (25.0%)	23 (28.8%)	
Living alone, *n* (%)	11 (13.8%)	13 (16.3%)	0.658 ^b^
Comorbidities			
# 0	43 (53.8%)	38 (47.5%)	0.510 ^b^
# 1–2	29 (36.3%)	36 (45.0%)	
# ≥ 3	8 (10.0%)	6 (7.5%)	
Hypertension	28 (35.0%)	21 (26.3%)	0.230 ^b^
Cardiac disease	8 (10.0%)	7 (8.8%)	0.786 ^b^
Diabetes mellitus	12 (15.0%)	5 (6.3%)	0.073 ^b^
Hyperlipidemia	7 (8.8%)	4 (5.0%)	0.349 ^b^
Osteoarthritis	4 (5.0%)	12 (15.0%)	**0.035** ^b^
History of falls, *n* (%)	11 (13.8%)	6 (7.5%)	0.200 ^b^
Time spent sitting (hour/day)	4 (3–6)	4 (3–6)	0.829 ^a^
Dietary habits, *n* (%)			
Vegan	—	2 (2.5%)	
Ovo-vegetarian	—	2 (2.5%)	
Lacto-vegetarian	—	4 (5.0%)	
Lacto-ovo-vegetarian	—	72 (90.0%)	
Omnivorous	80 (100%)	—	

Data are mean (95% CI), median (IQR), or frequency (percentage). BMI, body mass index; MMSE, mini mental state examination. Bold indicates significance with *p* < 0.05. ^a^ Mann–Whitney test; ^b^ Chi-square test or Fisher exact test.

**Table 2:** Physical activity and physical performance in vegetarians and nonvegetarians.

	Nonvegetarians (*n* = 80)	Vegetarians (*n* = 80)	*p-*Value	*p-*Value ^c^
Physical activity				
IPAQ-SF score (MET-min/week)	1155 (470–2942)	900 (477–1784)	0.105 ^a^	
Physical performance				
Handgrip strength (kg)	23.2 (20.2–26.4)	23.2 (20.6–26.0)	0.932 ^a^	
5xSTS (s)	7.2 (6.2–8.9)	7.4 (5.8–9.8)	0.895 ^a^	
Gait speed (m/s)	1.34 (1.28–1.39)	1.34 (1.28–1.40)	0.991 ^b^	0.563
TUG (s)	7.5 (6.5–9.6)	7.8 (6.7–9.0)	0.676 ^a^	
FRT (cm)	19.7 (18.7–20.6)	24.5 (23.2–25.8)	**<0.001** ^b^	**<0.001**

Data are mean (95% CI) or median (IQR). IPAQ-SF, international physical activity questionnaire-short form; MET, metabolic equivalent of task; TUG, timed up-and-go; FRT, functional reach test; 5xSTS, five-times-sit-to-stand. Bold indicates significance with *p* < 0.05. ^a^ Mann–Whitney test; ^b^ Independent *t*-test; ^c^ ANCOVA test.

**Table 3:** Numbers of vegetarians (*n* = 80) and nonvegetarians (*n* = 80) with low physical activity levels and poor physical performance.

	Nonvegetarians *n* (%)	Vegetarians *n* (%)	*p-*Value
Low Physical activity levels ^1^	22 (27.5%)	26 (32.5%)	0.490
Poor physical performance			
Slow gait speed (< 1.0 m/s)	9 (11.3%)	8 (10.0%)	0.797
TUG deficits (> 11 s)	11 (13.8%)	8 (10.0%)	0.463
FRT deficits (< 25.4 cm)	12 (15.0%)	6 (7.5%)	0.133
Low handgrip strength (< 18 kg)	8 (10.0%)	11 (13.8%)	0.463
5xSTS deficits (≥ 12 s)	6 (7.5%)	7 (8.8%)	0.772

TUG, timed up-and-go; FRT, functional reach test; 5xSTS, five-times-sit-to-stand. ^1^ Defined as IPAQ-SF score < 600 MET-min/week.

**Table 4:** Main reasons for low physical activity levels.

	Total (*n* = 48, 30%)	Nonvegetarians (*n* = 22)	Vegetarians (*n* = 26)	*p*-Value
Exhaustion after work	17 (35.4%)	8 (36.4%)	9 (34.6%)	0.936
Busyness (lack of time)	13 (27.1%)	5 (22.7%)	8 (30.8%)	
Lack of interest	10 (20.8%)	5 (22.7%)	5 (19.2%)	
knee pain	8 (16.7%)	4 (18.2%)	4 (15.4%)	

## Data Availability

The data used to support of this study are included with the article.
